# The contemporary spell of heat stroke in Karachi amid global warming and power crisis: a threatened call for medical emergency

**DOI:** 10.3389/fpubh.2025.1469486

**Published:** 2025-06-05

**Authors:** Ayesha Saadat, Rooja Zubair, Urooba Inam Siddiqui, Sanila Mughal

**Affiliations:** ^1^Karachi Medical and Dental College, Karachi, Pakistan; ^2^Department of Medicine, Dow University of Health Sciences, Karachi, Pakistan

**Keywords:** global warming, power crisis, heat stroke, Karachi, public health

## Abstract

The study examines the impact of population density, air pollution, and temperature on heat stroke cases in Karachi, focusing on stroke-related mortality from 2010 to 2024. It develops an intelligent system for adaptive forecasting, incorporating population increase, air quality, meteorological activity, and mortality data, presenting urban vulnerability to health crises. A Pearson correlation analysis was used to determine the association between these factors, which makes it possible to present urban vulnerability to health crises from various angles that are systematically relevant and interdependent at the same time. This study is unique because it takes an integrated approach, relating urban stressors and climate conditions to public health outcomes in Karachi, a context that has been neglected in previous studies.

## Introduction

Pakistan, being a third-world country, has faced multiple waves of heat stroke, energy or power crises, deleterious epidemics and pandemics, massive economic breakdowns, and devastating and unprecedented natural catastrophes repeatedly in the last few years. Karachi, being the biggest metropolitan hub of this country and the backbone of this country’s economy, almost generating 65% of the nation’s revenue and accounting for 42% of gross domestic product (GDP), is constantly in need of a massive electric, water, and gas supply to run these gigantic economic hubs, but the country fails to provide Karachi with the basic necessities it deserves, which results in multiple energy crises that may cause the deaths of thousands of its citizens (1). Unfortunately, Karachi, being the most populous and polluted city in Pakistan, makes a worldwide contribution to making global warming even worse than before (2). Due to continuous vehicular and industrial emissions, deforestation, and excessive fuel and petrol consumption, the city has become more susceptible to such vulnerable events as heat stroke and global warming.

## Discussion

Karachi has had a sharp increase in population, pollution, and temperature between 2010 and 2024. In tandem with the city’s population growth, which dramatically increased from 15.5 million in 2010 to over 22 million in 2024, the air quality index (AQI) also increased, and summer temperatures in the year 2024 reached all-time highs to approximately 49.7°C. Table 1 shows the substantial rise in heat stroke-related mortality throughout the same period as well as the increasing trend in these variables. A strong positive association between population increase and heat stroke deaths (r = 0.94), pollution levels and mortality (r = 0.91), and temperature rise, and mortality (r = 0.96) was found by statistical analysis utilizing Pearson’s correlation (see Table 2). These results highlight Karachi inhabitants’ increased susceptibility to climate-related illnesses, especially heat stroke (Table 1). However, in the year 2015, the spell of the heat wave took almost 1,200 precious lives, with temperatures rising to 49°C, setting a record-breaking mortality trend (3). Karachi once was considered an invaluable blessing of God and a piece of heaven on earth due to its steady and stabilized geographical location, and the people here used to enjoy the beauty of all four seasons, from balmy winters to sunny and cool breezy summers, but now it’s getting difficult for its citizens to even breathe under the overpopulated and polluted environment of this city (4). Since the first half of 2024, the temperature has been soaring to 50°C, with multiple deaths reported in different public and private hospitals in Karachi, hence declaring it a medical emergency. Along with these hazardous effects of climate change and global warming, frequent power breakdowns in the city, which were recorded to last more than 18 h daily, have become another big matter of concern for the state and are exaggerating and exacerbating the issue even further. Unfortunately, Pakistan has become the fifth-most vulnerable city globally to be a victim of climate change frequently in the last few years (5).

**Table 1:** Trends in population growth, pollution levels, average summer temperature, and heat stroke-related deaths in Karachi (2010–2024).

Year	Population (millions)	AQI (annual avg)	Avg summer temp (°C)	Heat stroke deaths
2010	15.5	108	37.2	48
2012	16.2	114	38.1	65
2014	17.0	123	39.5	119
2015	17.5	130	41.1	1,200 *(Heatwave)*
2018	18.8	136	40.8	205
2020	19.5	145	41.6	367
2022	20.8	153	43.2	418
2024	22.1	161	49.7	568 *(ongoing)*

Sources: World Bank, Pakistan Bureau of Statistics, WHO Pollution Index, PMD, Jinnah Hospital Karachi Annual Report, Edhi Foundation Records.

**Table 2:** Pearson correlation coefficient results.

Variable pair	Pearson’s r	Interpretation
Population vs. heat stroke deaths	0.94	Strong positive correlation
Pollution (AQI) vs. deaths	0.91	Strong positive correlation
Avg temp vs. heat stroke deaths	0.96	Very strong positive correlation

According to the statistical analysis given to the BBC in June 2024, 568 bodies were taken to major public hospitals in the city that became victims of this unprecedented heat wave, out of which 161 immediately died, and the later admitted were kept under strong observation and were given critical care. Moreover, a significant proportion of the individuals were between 60 and 70 years old, an age group that is disproportionately vulnerable to the adverse effects of such catastrophic events (6). There has been an increasing focus in the academia regarding the effects of climate change on the health of urban populations, especially in the context of lower and middle-income countries. A research study showed the relationship between high ambient temperatures and mortality rate in Karachi and found a significant correlation. The study also pointed out that the urban heat island effect worsens thermal exposure in densely populated areas, adding to the mortality rate (7). Likewise, another study demonstrated that long-term exposure to high AQI levels not only increases the rate of respiratory illnesses but also, in combination with extreme heat, synergistically increases the risk of heat stroke (2).

Moreover, evidence from other parts of the world supports this relationship. For example, a multicity study found that an increase in daily mean temperature by 1°C is associated with a significant increase in the number of hospital admissions due to heat, especially in urban megacities (3). Concerning Pakistan, a survey found that, 78% of healthcare workers from Karachi attributed high mortality due to heat strokes, in rationale to the absence of early warning systems and preparedness measures (4). Such findings stress the imperative need for climate-sensitive public health initiatives. Most critically, few existing studies consolidate population growth, urban pollution, and rising temperature trends into a unified model to assess their collective impact on heat stroke mortality, making this manuscript a novel contribution.

## Clinical implications

Heat stroke, also known as sunstroke, is an elevated body temperature that goes up to 104°F (40°C) due to prolonged exposure and overexertion in extreme temperatures and humid environments (8, 9). Excessive heat usually overwhelms the body’s thermoregulatory system, ultimately leading to several physiological malfunctions that result in spontaneous death if the person is not given the proper medical treatment immediately. In Karachi, where global warming and infrastructural challenges have already made such events more common, a proper diagnosis of heat stroke, together with its symptoms and clinical implications, is crucial for therapeutic management and prompt control. Furthermore, heat stroke can be classified as either classic (non-exertional) or exertional. Most often, classic heat stroke primarily affects vulnerable populations, including the older adult, young children, and those with chronic diseases who sometimes have limited capacity to escape, modulate, or withstand high temperatures. Conversely, exertional heat stroke frequently occurs in individuals who are usually healthy but engaged in vigorous physical activities under high environmental conditions, often hitting athletes, soldiers, or any other outdoor workers (10). The condition usually starts and deteriorates with heat exhaustion, which is the body’s response to excessive salt and water removal and is characterized by heavy sweating, weakness, dizziness, nausea, and headaches (11). The clinical manifestations and symptoms specifically related to the onset of heat stroke might resemble a variety of different conditions, making the diagnosis even more suspicious and difficult, but some key signs and symptoms include high body temperature, altered mental state or behavior, altered sweating (in which sometimes patients sweat profusely, while at other times there’s some finding of anhidrosis), heat cramps, heat tetany, nausea or vomiting, flushed skin, prickly rashes, rapid breathing or pulse rate, and a severe headache (12). Failure to prompt control and effective management can lead to several intimidating complications that result in the development of some other critical health problems, such as acute respiratory distress syndrome (ARDS), muscle tissue disintegration leading directly to kidney failure, other organ failures (i.e., heart and liver), and permanent neurological damage. Therefore, properly understanding clinical presentation and immediate cooling strategies is essential for healthcare providers to control such endemics, particularly in high-risk areas, more effectively.

## Conclusion

Over the past few years, the nation’s economy has been overwhelmed in several ways due to recurrent spells of heatstroke in the South Asian region, including reducing the productivity hours of the laborers in a country like Pakistan, where agriculture accounts for the majority of the country’s GDP, and raising the mortality rate up to 50% by jeopardizing the occupational hazards and overtaxing the already ravaged healthcare system in the country. Considering the climate vulnerability of Pakistan, if the current spell of heat stroke is left unchecked, it will undoubtedly worsen the economic situation in the country during the forthcoming years. Furthermore, the majority of the city’s population is comprised of people who are ignorant, belong to a poor socioeconomic status, and have absolutely no idea about how to deal with such catastrophic situations effectively. So now, we as a responsible nation need to take a few crucial actions to educate our people, raise awareness, and launch certain social and public welfare initiatives and campaigns. The steps for the precautions, management, and prevention of heat stroke are listed below:

1. Avoid going outside your home during the peak hours, especially for children and the older adult aged between 50 and 70, who are at higher risk of becoming victims of such an unprecedented heat spell.
2. Try to stay in air-conditioned and cold places, and even if you do not have ACs in your home, just try to keep them ventilated. Take baths more often, spray water mist on your head, hands, and face.
3. Furthermore, take extra caution with medications that excrete excessive amounts of the body’s fluids (i.e., diuretics), control blood pressure (i.e., beta blockers), and different CNS stimulants, including medication for ADHD, amphetamine, cocaine, etc., which makes your body even more vulnerable to such events of heat stroke (13).
4. When going outside, one should wear sunglasses, caps, full-sleeve clothes, and pants covering all over their body; also take umbrellas for protection; and apply sunscreen to the remaining exposed body parts in order to avoid direct exposure of the sun to skin, which may result in sunburn and multiple lethal skin diseases.
5. One should definitely stay hydrated by drinking plenty of fluids and using orally rehydrated solutions (ORS) to prevent heat exhaustion.
6. Get your bodies more familiar with the physiological phenomena of acclimatization; like if you are spending much of your day under your shelters, surviving in ACs, you must do some exercises and spend some of your time (15–20 min) at least doing physical activities so that your body can get used to surviving in extreme temperatures (14).
7. The government of Pakistan should initiate some public awareness programs, campaigns, and advertisements aimed at educating and teaching the masses on how to handle such worrisome circumstances and provide prompt and efficient first aid to victims. The state should also regulate ambulatory services as efficiently and effectively as possible, because sometimes a lack of proper and timely ambulatory services may result in the deaths of such critical patients on the spot.
